# Neutrophil-to-Lymphocyte Ratio Is Independently Associated With Severe Psychopathology in Schizophrenia and Is Changed by Antipsychotic Administration: A Large-Scale Cross-Sectional Retrospective Study

**DOI:** 10.3389/fpsyt.2020.581061

**Published:** 2020-10-30

**Authors:** Xia Zhou, Xiaolan Wang, Rui Li, Jun Yan, Ying Xiao, Weiguang Li, Hong Shen

**Affiliations:** ^1^Neuro-Psychiatric Institute, Nanjing Medical University Affiliated Brain Hospital, Nanjing, China; ^2^Department of Psychiatry, Nanjing Medical University Affiliated Brain Hospital, Nanjing, China; ^3^School of Pharmacy, Nanjing Medical University, Nanjing, China; ^4^Department of Geriatric Neurology, Nanjing Medical University Affiliated Brain Hospital, Nanjing, China; ^5^College of Science, China Pharmaceutical University, Nanjing, China; ^6^College of Life Science and Technology, China Pharmaceutical University, Nanjing, China

**Keywords:** neutrophil-to-lymphocyte ratio, antipsychotics, brief psychiatric rating scale, clinical global impression severity scale, schizophrenia

## Abstract

**Background:** Immunological and inflammatory mechanisms play an important role in schizophrenia. The neutrophil-to-lymphocyte ratio (NLR) is a value obtained by dividing the absolute number of neutrophils by the absolute lymphocyte count and represents a biomarker of systemic inflammatory response. There are studies investigating NLR association with psychopathology. However, the relationship has been only studied in small numbers of patients with schizophrenia, which leads to conflicting results and makes the meta-analytic data difficult to interpret. The aim of this study is to perform large-scale cross-sectional analysis on the potential correlation between NLR and disease severity in schizophrenic patients with or without medication.

**Methods:** This cross-sectional retrospective study was conducted in Nanjing Medical University Affiliated Brain Hospital. We identified inpatients with schizophrenia between July 12, 2018 and March 27, 2019 and collected data of NLR, the Clinical Global Impression Severity scale (CGI-S) score and the Brief Psychiatric Rating Scale (BPRS) score.

**Results:** The records of 1,144 identified patients (10.8% drug-free patients) were analyzed. We found that NLR was significantly decreased in schizophrenic patients after antipsychotic administration and there was the discrepant correlation between NLR and psychiatric symptoms in patients with or without antipsychotic medication. The results of multivariate logistic regressions showed that NLR was positively associated with the severity of disease (i.e., the CGI-S score and the BPRS total score) in drug-free patients, and it was negatively associated with the BPRS negative symptoms (i.e., the BPRS negative symptoms score) in drug-therapy patients.

**Conclusion:** The study is the first to confirm the hypothesis that NLR is independently associated with severe psychopathology in schizophrenia and is changed by antipsychotic administration.

## Introduction

Schizophrenia is a complex and multifactorial mental disorder mainly characterized by a wide scope of psychotic symptomatology, such as anxiety, emotional withdrawal, conceptual disorganization, guilt feelings, grandiosity, depressive mood, hostility, suspiciousness, hallucinatory behavior, motor retardation, unusual thought content, blunted affect, and excitement (1). A growing body of evidence supports the existence of immune system abnormalities in schizophrenia. There are central and peripheral immune and inflammatory biomarker alterations in numerous neuropsychiatric conditions (2). Nevertheless, not many studies have been conducted on which immunological biomarker alterations may affect schizophrenic symptoms.

Abnormality of immune cells in circulation may be involved in schizophrenia (3, 4). For example, the white blood cells (WBCs, leucocytes) that form the peripheral immune system and are crucial in inflammatory processes were initially found to have abnormal changes in patients with schizophrenia over half of a century ago (5, 6). Significant correlations were found between WBCs and the Brief Psychiatric Rating Scale (BPRS) total score, negative symptoms score, as well as anxious depression factor score in patients with non-affective psychosis (7). Thus far, it was observed that there were possible differences between normal and schizophrenic subjects in the levels of subtypes of leukocytes, especially neutrophil and lymphocyte. However, both of these factors have inconsistent results about their association with psychiatric symptoms. For example, neutrophil count was described to be increased in schizophrenia (8). The recent study reported that neutrophil count was associated with the PANSS total score in first-episode psychosis (9). These results were not replicated in the study of Garcia-Rizo et al. (10). Moreover, a meta-analysis of blood lymphocytes noted that absolute levels of total lymphocytes were significantly increased in drug-naïve first-episode psychosis, but there was no report of the association of lymphocytes with psychiatric symptoms (11).

The neutrophil-to-lymphocyte ratio (NLR) is a value obtained by dividing the absolute number of neutrophils by the absolute lymphocyte count and represents a biomarker of systemic inflammatory response. NLR may reflect the combined prognostic information of these two processes and be a stronger predictor of the outcome than either alone. It has received more attention and been used to determine seriousness in many diseases, e.g., coronary artery diseases, malignancies, diabetes mellitus and appendicitis (12, 13). In recent years, several studies reported increased NLR in schizophrenia (14–16). A meta-analysis of eight studies (including 683 patients and 551 healthy controls) showed that subjects with non-affective psychosis had a significantly higher NLR and monocyte-to-lymphocyte ratio (MLR) compared with healthy controls (17). Another meta-analysis of 10 studies (804 schizophrenic patients, 671 controls) found that NLR in patients with schizophrenia was increased, both in chronic disease and in first-episode psychosis (18). According to the information, it had been hypothesized that NLR was positively correlated with psychotic symptoms. However, small numbers of patients with schizophrenia led to inconsistent findings, making the meta-analytic data difficult to interpret. Kulaksizoglu et al. documented the significant positive relationships of the Positive and Negative Syndrome Scale (PANSS) total subscale to NLR (19). In contrast, data from three studies provided the evidence that there was no significant relationship between NLR and disease severity as manifested in clinical scores [e.g., the BPRS, the PANSS, and the Clinical Global Impression Severity scale (CGI-S)] in patients with schizophrenia (15, 20, 21). Bustan et al. also found NLR was not significant correlate to psychopathology in non-affective psychotic adolescent inpatients (14). Furthermore, the meta-analysis of Karageorgiou et al. (18) reported that a differing PANSS positive symptom score did not appear to alter the NLR mean difference due to insufficient data (The average sample size of individual studies was 64 schizophrenic patients and 65 controls, *n* = 3). Moreover, they could not evaluate the effect of negative symptomatology (e.g., PANSS negative symptoms) or general mental condition (e.g., BPRS) on NLR. As such, data from the large studies are important.

Theoretically, antipsychotic treatment could alleviate psychiatric symptoms and exert anti-inflammatory effects, and therefore it is possible that antipsychotic treatment also affects the relationship between NLR and disease severity. However, only a handful of studies with small sample sizes have investigated the association between antipsychotic treatment and NLR (15, 21). Neither study reported a significant effect of psychotropic medications on NLR in patients with schizophrenia. In a meta-regression analysis (549 patients and 626 healthy controls, *n* = 7), a modest but significant effect of antipsychotics was noted. This finding implies that antipsychotic medicated subjects may exhibit a higher NLR irrespective of a schizophrenia diagnosis (18). Of note, no study has evaluated the potential association between NLR and disease severity in schizophrenic patients with or without antipsychotic medication.

This study is therefore to perform a large-scale cross-sectional study to evaluate the potential correlation between NLR and disease severity in patients with schizophrenia, particularly, whether NLR is correlated with discrepant psychiatric symptoms in patients with or without antipsychotic medication.

## Methods

### Patients and Study Design

We used data from patients with schizophrenia who had undergone a physical examination in Nanjing Medical University Affiliated Brain Hospital between July 12, 2018 and March 27, 2019. The inclusion criteria in the study were as follows: (1) inpatients with the diagnosis of schizophrenia, any subtype, as evaluated by two independent experienced psychiatrists according to the International Classification of Diseases (ICD10); and (2) patients with schizophrenia who had taken no antipsychotics or were off psychiatric medication for at least 1 month before blood sampling (the drug-free patients), as well as received antipsychotic therapy for at least 1 week (the drug-therapy patients). All participants were Han Chinese. The exclusion criteria were as follows: (1) aged <18 or >60 years; (2) has an ongoing hematologic, cardiovascular, hepatic, renal disease, diabetes mellitus, hypertension, hyperlipidemia, systemic disorders known to be associated with immunological abnormalities, thyroid disease, acute infection, history of severe head injury and surgery, or malignancy, according to medical diagnoses; (3) currently pregnant or breastfeeding; (4) no data available for outcomes.

For all participants, the data of demographics and laboratory measurements including neutrophil and lymphocyte counts were noted retrospectively, and NLR was calculated from these. For inpatients with schizophrenia, we used the electronic medical record system of Nanjing Medical University Affiliated Brain Hospital to collect patients' clinical data including sex, age, medical diagnoses, age of illness onset, and illness duration. The BPRS score and CGI-S score were assessed by two independent experienced psychiatrists. For all laboratory measurements, laboratory personnel were blinded to clinical information. All data were collected by two independent observers, without any knowledge of this study. The study protocol was reviewed and approved by the ethics committee of Nanjing Medical University Affiliated Brain Hospital. The study was based on data with routine clinical care and administration. All patients were not allowed to smoke and consume alcohol during hospitalization.

### Brief Psychiatric Rating Scale (BPRS)

The symptoms of schizophrenia were assessed using the BPRS. The present version of the instrument contains 18 items that assess the following symptoms: somatic concern, anxiety, emotional withdrawal, conceptual disorganization, guilt feelings, tension, mannerisms and posturing, grandiosity, depressive mood, hostility, suspiciousness, hallucinatory behavior, motor retardation, uncooperativeness, unusual thought content, blunted affect, excitement, and disorientation. The items are administered by a clinician based on a 7-point scale ranging from 1 (not present) to 7 (extremely severe) with total scores ranging from 18 to 126, with the higher scores representing greater severity of symptoms (22). The 18 items are composed of five subscale scores: affect (anxiety, guilt, depression, somatic), positive symptoms (unusual thought content, conceptual disorganization, hallucinatory behavior, grandiosity), negative symptoms (blunted affect, emotional withdrawal, motor retardation), resistance (hostility, uncooperativeness, suspiciousness), and activation (excitement, tension, mannerisms and posturing) (23). The categories of the BPRS total score were classified as followed: 18–31 = not or mildly ill; 32–53 = moderately ill; and 54–126 = severely ill (24).

### Clinical Global Impression Severity Scale (CGI-S)

The CGI-S was also available for the evaluation of the severity of disease with the clinician's impression of the patient's current illness state, and ranged from 1 to 7, with the higher scores representing greater severity of disease (25).

### Statistical Analyses

The demographic and clinical characteristics of patients with schizophrenia were checked for data normality using the Shapiro-Wilk test. Descriptive analyses included the calculations of mean [standard deviation (SD)] or median [interquartile range (IQR)] for continuous variables, as appropriate, and frequencies and percentages for categorical variables. The demographic characteristics, blood count parameters, and psychiatric symptom scores between patients with and without antipsychotic administration were compared with the chi-square test or the Fisher exact test for categorical variables, as appropriate, and the Kruskal-Wallis test for continuous variables.

Using spearmen correlation coefficients, we assessed potential associations between NLR in a continuous fashion and BPRS scores, as well as other variables, such as age, sex, age of illness onset, illness duration, and antipsychotic administration. Sex [male (0) and female (1)] and antipsychotic administration [drug-free (0) and drug-therapy (1)] were denoted nominally; other variables were denoted in a continuous fashion. These analyses were undertaken for the total group, as well as across categories of antipsychotic administration subgroups.

Multivariate logistic regression analyses were used to evaluate the associations between NLR as a continuous independent variable and categorical dependent variables, including the CGI-S score, the BPRS total score and the five subscale scores when other categorical and/or continuous independent variables such as sex, age, age of illness onset, illness duration, neutrophil count, lymphocyte count, antipsychotic administration, and their interactions were taken into consideration. The categorical dependent variables were divided into trisections, with the first trisection serving as the referent category. The categories of the CGI-S score and the five subscale scores were discriminated by ROC curve analysis. The sex and antipsychotic administration were categorical variables, with male sex and drug-therapy serving as the referent categories. After the logistic regression analysis of the whole study population, we conducted the logistic regression stratified by antipsychotic administration to further examine the possible association of NLR and the clinical manifestation. The model in the entire study population was constructed using forced entry of all co-variables, and other models in the subgroups were used entry stepwise elimination, including variables with *p* < 0.10. We used ORs [95% confidence intervals (CIs)] to report the results of logistic regression.

Before running the logistic regression analyses, the collinearity was checked by the variance inflation factor (VIF) that indicates how much the variance of the coefficient estimate is inflated by multicollinearity. The VIF values of >2.5 were considered as high collinearity (26). ROC curves were created by plotting the range of sensitivity and specificity pairs for the CGI-S and BPRS subscale scores, with BPRS total score categories (the BPRS total: ≤31 vs. >32 or ≤53 vs. >54) as the classifier variable. To determine the threshold of the subscale scores, the Youden index was used to discriminate between patients (the BPRS total: >32 or >54) and controls (the BPRS total: ≤31 or ≤53). When the Youden index reached the maximum value, the corresponding CGI-S score and BPRS subscale scores were considered the optimum cut-off threshold. Then, the cut-off thresholds of the subscale scores were defined.

All *p* values were two-tailed. The significance level was set at 0.05. The statistical analyses were performed with the Statistical Package for Social Sciences (SPSS, version 19; IBM, Armonk, NY, USA).

## Results

**Figure 1:**
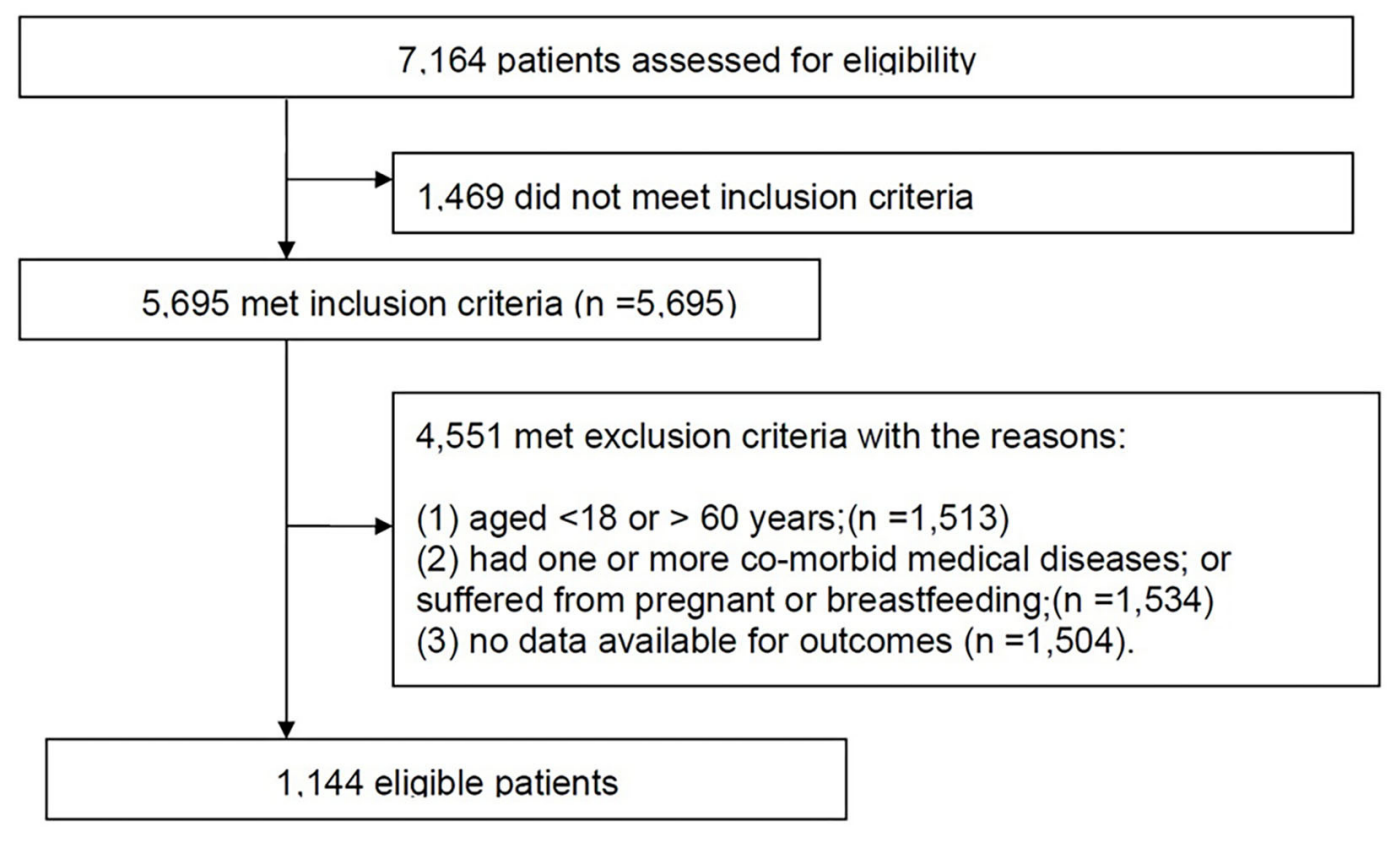
The study inclusion and exclusion process.

The data of the retrospective study were from Nanjing Medical University Affiliated Brain Hospital between July 12, 2018 and March 27, 2019. A total of 7,164 patients were identified from the initial search. The study inclusion and exclusion processes are shown in Figure 1. Finally, 1,144 eligible patients with schizophrenia had available data for analysis (Table 1). The characteristics for those patients with or without antipsychotic administration are provided. As depicted, patients without antipsychotic administration were more likely to have a higher NLR (1.79 vs. 1.01, *p* < 0.001), a lower lymphocyte count (2.06 vs. 2.86, *p* < 0.001), and a higher neutrophil count (4.27 vs. 3.55, *p* < 0.001). Psychopathologically, patients without antipsychotic administration have higher CGIS score (5 vs. 4, *p* < 0.001) and BPRS total score (49 vs. 37, *p* < 0.001).

**Table 1:** Demographic and clinical characteristics of the patients with schizophrenia.

	**Total (*n* = 1,144)**	**Total (*****n*** **=** **1,144)**		
		**Drug-free (*n* = 123)**	**Antipsychotic therapy (*n* = 1,021)**	***p***
Age, year	35 (27–45)	34 (28–43)	35 (27–45)	0.549
Male sex, *n* (%)	371 (32.4)	32 (26.0)	343 (33.6)	**0.036**
Age of illness onset, year	23 (18–28)	24 (20–32)	23 (18–28)	**0.001**
During of illness, year	10 (5–17)	6 (3–12)	10 (5–17)	** <0.001**
Drug administration				
No, *n* (%)	123 (10.8)	–	–	–
Yes, *n* (%)	1,021 (89.2)	–	–	–
Clozapine, *n* (%)	74 (6.5)	–	–	–
Aripiprazole, *n* (%)	77 (6.7)	–	–	–
Amisulpride, *n* (%)	117 (10.2)	–	–	–
Olanzapine, *n* (%)	171 (14.9)	–	–	–
Risperidone, *n* (%)	144 (12.6)	–	–	–
Quetiapine, *n* (%)	17 (1.5)	–	–	–
Chloropromazine, *n* (%)	2 (0.2)	–	–	–
Ziprasidone, *n* (%)	5 (0.4)	–	–	–
Perphenazine, *n* (%)	5 (0.4)	–	–	–
Antipsychotic Combinations, *n* (%)	409 (35.8)	–	–	–
Lymphocyte count, 10^9^/L	2.73 (1.82–5.83)	2.06 (1.55–3.64)	2.86 (1.87–5.98)	** <0.001**
Neutrophil count, 10^9^/L	3.60 (2.77–4.75)	4.27 (3.18–5.98)	3.55 (2.73–4.55)	** <0.001**
NLR	1.08 (0.60–1.90)	1.79 (0.85–2.98)	1.01 (0.60–1.80)	** <0.001**
CGIS score (continuous)	4 (3–5)	5 (5–6)	4 (3–5)	** <0.001**
BPRS total score (continuous)	38 (30–47)	49 (45–56)	37 (30–45)	** <0.001**
BPRS affect score (continuous)	5 (4–8)	7(4–9)	5 (4–7)	** <0.001**
BPRS positive symptoms score (continuous)	8 (6–11)	10 (8–13)	8 (6–10)	** <0.001**
BPRS negative symptoms score (continuous)	6 (4–8)	7 (5–10)	6 (4–8)	** <0.001**
BPRS resistance score (continuous)	7 (5–10)	12 (9–15)	7 (5–10)	** <0.001**
BPRS activation score (continuous)	5 (3–6)	6 (5–8)	4 (3–6)	** <0.001**

*When p < 0.05, the corresponding values are bold*.

Spearman correlation coefficients were used to assess the association between NLR and potential confounding factors in schizophrenia preferences as shown in Table 2. Overall, NLR was significantly associated with the CGI-S score (*r* = 0.099, *p* = 0.001), the BPRS negative symptoms score (*r* = −0.062, *p* = 0.036) and the BPRS resistance score (*r* = 0.063, *p* = 0.033). There was a negative relation between NLR and antipsychotic medication in total (*r* = −0.193; *p* < 0.001). Then, in the drug-free subgroup, NLR was significantly associated with the CGI-S score (*r* = 0.296, *p* = 0.001) and the BPRS activation score (*r* = 0.272, *p* = 0.002); whereas, in the drug-therapy subgroup, NLR was negatively related to the BPRS negative symptoms score (*r* = −0.111, *p* < 0.001).

**Table 2:** The spearman correlations analysis between NLR and CGIS and BPRS scores, and other variables.

	**Total (*****n*** **=** **1,144)**		**Drug-free (*****n*** **=** **123)**		**Antipsychotic therapy (*****n*** **=** **1,021)**	
	***R***	***p***	***R***	***p***	***R***	***p***
Age	**−0.262**	** <0.001**	0.023	0.797	**−0.290**	** <0.001**
Sex	–0.012	0.693	–0.050	0.584	–0.019	0.551
Age of illness onset	**0.115**	** <0.001**	**0.227**	**0.011**	**0.071**	**0.023**
During of illness	**−0.513**	** <0.001**	–0.172	0.057	**−0.505**	** <0.001**
CGIS score	**0.099**	**0.001**	**0.296**	**0.001**	0.005	0.882
BPRS total score	0.040	0.175	0.176	0.052	–0.045	0.154
BPRS affect score	0.037	0.206	0.099	0.277	–0.002	0.956
BPRS positive symptoms score	0.035	0.237	0.094	0.300	–0.011	0.727
BPRS negative symptoms score	**−0.062**	**0.036**	0.042	0.648	**−0.111**	** <0.001**
BPRS resistance score	**0.063**	**0.033**	0.146	0.106	–0.012	0.699
BPRS activation score	0.047	0.109	**0.272**	**0.002**	–0.018	0.567
Drug administration	**−0.193**	** <0.001**	–	–	–	–

*When p < 0.05, the corresponding values are bold*.

To interpret the regression analyses, we had to (i) ascertain that there was no sign of multicollinearity and (ii) define the cut-off thresholds of the CGI-S score and BPRS subscale scores. First, with regard to checking for collinearity, the VIF values of other variables ranged from 1.077 to 1.492 and only that of age and NLR was more than 2.5. Thus, there was multicollinearity between NLR and age. Second, based on the results of ROC curve analyses, the cut-off values and the categories of the CGI-S score and BPRS subscale scores were determined.

We then investigated the association of NLR with psychiatric symptoms and examined whether there was an association between NLR and severe psychiatric symptoms in schizophrenia on multivariate analysis controlling for other variables and the interactions of NLR and age (Table 3). Unfortunately, we did not find that NLR was significantly associated with the CGI-S score, the BPRS total score and any of BPRS subscale scores in the whole study population.

**Table 3:** Logistic regression analyses of NLR associated with CGIS or BPRS scores after controlling for other variables.

	**Total (*****n*** **=** **1,144)**		**Drug-free (*****n*** **=** **123)**		**Antipsychotic therapy (*****n*** **=** **1,021)**	
	**OR (95% CI)**	***P***	**OR (95% CI)**	***p***	**OR (95% CI)**	***p***
**CGIS score**						
Moderate (4)^a^	0.795 (0.508–1.244)	0.315	**63.578 (2.578**–**1,567.894)**	**0.011**	–	–
Severe (5–7)^a^	0.707 (0.447–1.117)	0.137	**53.617 (2.180**–**1,318.767)**	**0.015**	–	–
**BPRS total score**						
Moderate (32–53)^b^	1.295 (0.772–2.175)	0.327	4.049 (0.973–16.843)	0.055	–	–
Severe (54–126)^b^	1.344 (0.701–2.577)	0.373	**4.312 (1.035**–**17.931)**	**0.045**	–	–
**BPRS affect score**						
Moderate (6)^c^	0.966 (0.544–1.718)	0.907	–	–	–	–
Severe (7–28)^c^	0.999 (0.657–1.520)	0.996	–	–	–	–
**BPRS positive symptoms score**						
Moderate (7–10)^d^	1.245 (0.763–2.034)	0.380	–	–	–	–
Severe (11–28)^d^	0.754 (0.430–1.324)	0.326	–	–	–	–
**BPRS negative symptoms score**						
Moderate (6)^e^	0.596 (0.323–1.100)	0.098	–	–	1.058 (0.911–1.230)	0.459
Severe (7–21)^e^	0.886 (0.601–1.307)	0.542	–	–	**0.850 (0.742**–**0.973)**	**0.018**
**BPRS resistance score**						
Moderate (7–9)^f^	1.053 (0.639–1.737)	0.838	3.412 (0.829–14.049)	0.089	–	–
Severe (10–21)^f^	0.911 (0.553–1.501)	0.714	3.526 (0.861–14.440)	0.080	–	–
**BPRS activation score**						
Moderate (5–6)^g^	0.869 (0.517–1.462)	0.598	–	–	–	–
Severe (7–21)^g^	1.337(0.797–2.242)	0.271	–	–	–	–

a *CGIS score: not or mild (1–3) as the referent category*.

b *BPRS total score: not or mild (18–31) as the referent category*.

c *BPRS affect score: not or mild (4–5) as the referent category*.

d *BPRS positive symptom score: not or mild (4–6) as the referent category*.

e *BPRS negative symptom score: not or mild (3–5) as the referent category*.

f *BPRS resistance score: not or mild (3–6) as the referent category*.

g *BPRS activation score: not or mild (3–4) as the referent category*.

*When p < 0.05, the corresponding values are bold*.

To assess associations of NLR with psychiatric symptoms in the different subgroups, the results of logistic regression stratified by antipsychotic administration are shown in Table 3. In the drug-free subgroup, NLR was positively associated with worse psychiatric symptoms, both the CGI-S score (moderately ill: OR: 63.578, *p* = 0.011; severely ill: OR: 53.617, *p* = 0.015) and the BPRS total score (moderately ill: OR:4.049, *p* = 0.055; severely ill: OR: 4.312, *p* = 0.045) and there was a marginal correlation between NLR and worse BPRS resistance (moderately ill: OR:3.412, *p* = 0.089; severely ill: OR: 3.526, *p* = 0.080). In the drug-therapy subgroup, there was a negative correlation between NLR and severer negative symptoms (severely ill: OR: 0.850, *p* = 0.018).

## Discussion

The present large-scale study is the first to demonstrate an independent association of NLR with the severity of disease in schizophrenia and its potential change after antipsychotic administration intervention. We found that NLR was significantly higher in schizophrenic patients who were not taking antipsychotic medication and there was the discrepant correlation between NLR and psychiatric symptoms in schizophrenic patients with or without antipsychotic therapy. After stratification by antipsychotic administration, NLR was positively associated with the severity of disease (i.e., the BPRS total score and the CGI-S score) in drug-free patients, whereas it was negatively associated with negative symptoms (i.e., the BPRS negative symptoms score) in drug-therapy patients. These findings remained significant after controlling for potential confounding factors including sex, age, age of illness onset, illness duration, neutrophil count, lymphocyte count, and the interaction of NLR and age.

NLR is an important indicator of inflammation and it is easily measured, reproducible, and inexpensive for routine use. There are a few studies assessing the relationship between NLR and psychopathological symptoms in schizophrenia. Our present results are not consistent with those of the previous literatures (14, 15, 19, 21). Apart from small sample sizes, the conflicting results have been taken place due to heterogeneous patient populations and methodological discrepancies. First, our findings presented that NLR levels were decreased after antipsychotic intervention. And there was a negative correlation between NLR and antipsychotic medication in total patients. It is indicated that there is a significant effect of antipsychotics on NLR in patients with schizophrenia. Thus, it is necessary to distinguish drug-free and drug-therapy patients from heterogeneous patient populations. Second, the collinearity among the explanatory variables in the regression models may cause indeterminate parameters, infinitely large standard errors of the estimates, and then statistically insignificant coefficients. To control for the presence of collinearity, we computed the VIF values of the explanatory variables. The results indicated that there was multicollinearity between NLR and age. Then, the interaction between NLR and age was entered the regression models as a potential confounding factor. Third, these results of previous literatures were gained by Spearman or Pearson correlation analyses. In theory, Spearman or Pearson correlation tests can be used to assess whether two variables have a linear relationship with each other, but these correlation analyses cannot avoid the interference of the potential confounding. Hence, we conducted logistic regression analyses to assess the association of NLR and psychiatric symptoms after controlling for other variables. In summary, we find that NLR is positively associated with the severity of disease in drug-free patients with schizophrenia.

The explanations about the association between NLR and the severity of disease are complex. The most common one is the immunological roles in schizophrenia. NLR reflects neutrophil-dependent and/or lymphocyte mediated immune responses. Immune responses of the two immune cells are likely relative to the manifestation of schizophrenia symptoms. A few direct autopsy studies reported that the increased number of lymphocytes in the brain parenchyma or hippocampus of patients with schizophrenia (27, 28). Of note, Busse et al. (29) distinguished between residual (prevailing negative symptoms) and paranoid (prominent positive symptoms) schizophrenia, and reported that in the posterior hippocampus, higher densities of CD3+ T-lymphocytes and CD20+ B-lymphocytes were observed in residual vs. healthy controls (CD3: left: *p* = 0.057, right: *p* = 0.069; CD20: left: *p* = 0.008, right: *p* = 0.006). It was indicated that blood-brain barrier (BBB) impairment and infiltration of T cells and B cells may contribute to the pathophysiology of residual schizophrenia (prevailing negative symptoms). Regarding neutrophils, clinical autopsy evidence suggested that neutrophils migrate into the brain and exert destructive actions in the cerebral tissue in neurodegenerative diseases, including Alzheimer's disease (AD) and multiple sclerosis (MS) (30). Although schizophrenia is not a neurodegenerative disease, it may involve some neurodegenerative processes; not only dementia but also schizophrenic patients have a significant volume reduction of some specific regions in the brain (31). Núñez et al. supplied the indirect evidence of brain tissue loss associated with neutrophils in psychosis by a high-resolution T1-weighted structural image. Higher neutrophil count was associated with more severe clinical symptomatology (9). Thus, it is speculated that neutrophils and/or lymphocytes-mediated immune responses in brain play an important role on the clinical manifestation in schizophrenia.

Currently, scarce studies have the evidence of the effects of antipsychotic medication on NLR. In a large study, NLR was significantly higher in first episode schizophrenic patients who were not taking antipsychotic medication (20). In our study, we also found that NLR was significantly lower in drug-therapy patients than drug- free patients. NLR decrease was due to the significant decrease in the number of neutrophils and increase in the number of lymphocyte counts. The marketed antipsychotics, like clozapine, chlorpromazine and pimozide, could reduce the neutrophil count (32). In 1977, eight fatal cases of clozapine-induced agranulocytosis were observed in Finland (33). Clozapine-induced neutropenia occurs in ~1% of patients. In recent decades, it was found that patients with schizophrenia who take other antipsychotic drugs, including flupenthixol, haloperidol, thioridazine, trifluoperazine, olanzapine, risperidone, and sulpiride also had a risk of developing neutropenia (34). Notable, neutrophils are mainly known for their pro-inflammatory role in anti-bacteria. Recent data show that novel neutrophil functions have emerged in addition to their classical anti-microbial role. One of these functions is the suppression of T cell responses. For example, one important sub-population of circulating neutrophils is the granulocytic myeloid derived suppressor cells (G-MDSC) that has been shown to directly suppress T cell functions in a contact-dependent manner (35). Suppressive neutrophils are involved in T cell-mediated responses. The *in vitro* study reported that the CD62L^dim^ hypersegmented neutrophils potently suppressed T cell proliferation in dose-dependent manner within range of 1:10–2:1 NLR (36). Hence, we speculate that antipsychotics may down-regulate the suppression of T cell activation and proliferation through reducing the number of neutrophils. Consequently, antipsychotic medication presented the effects on NLR, which is a likely reason that antipsychotic medication alleviates psychiatric symptoms.

Interesting, it was found that in drug-therapy patients, NLR was negatively related to negative symptoms (*r* = −0.111, *p* < 0.001); and it remained significantly associated with severe negative symptoms (OR: 0.850, *p* = 0.018) after controlling for potential confounding factors. That is, for every 1-unit decrease in NLR, there was a 17.6% increased risk of severe negative symptoms. Generally, negative symptoms refer to poor emotional reactions or thought processes, including emotion impoverishment, speech barrier, thought inflexibility, and decreased activity. Essentially clinically effective antipsychotics are dopamine (DA) antagonists and target dopamine receptors (DRs). The administration of DA antagonists for disease treatment potentially causes the manifestation of secondary negative symptoms, in particular, akinesia or bradykinesia (37–39). In Fact, DRs are not only expressed in the CNS, but also expressed on the surface of peripheral immune system cells, including lymphocytes and neutrophils (40–42). The characteristics of peripheral lymphocytes in schizophrenia might reflect the clinical manifestation. Kwak et al. (42) ascertained that the patients with the higher D3R (D2-like) expression of peripheral lymphocytes had statistically and significantly high BPRS score in the drug-free and drug-naïve patients. However, the administration of DA antagonists for disease treatment potentially causes decreased sensitivity of DRs. It was observed that D3DR mRNA expression of peripheral lymphocytes in drug-medicated schizophrenics was significantly reduced compared to drug-free schizophrenics (42). Of note, as DA antagonists, antipsychotic drugs are more likely to over-occupy D2 receptor, to diminish receptor expression and to cause decreased sensitivity of DRs and secondary negative symptoms. This reason may explain that NLR is negatively related to negative symptoms in drug-therapy patients.

The limitations of this study are as follows: First, this study included 123 drug-free patients and 1,021 drug-therapy patients. Since the relatively small sample size of drug-free patients may have introduced potential bias in analyses of the total group, we put more attention on the results of subgroups. Second, the different sample sizes between male and female patients were likely to another potential bias. Nevertheless, our results presented that there was no sex difference in NLR. So we ignored the different sample sizes between the two sexes. Third, because a meta-analysis has revealed that the increased NLR is found in patients with both chronic-type schizophrenia and first-episode psychosis (18), we did not discriminate between patients with acute psychotic state and those in clinical remission.

In conclusion, the present large-scale study is the first to demonstrate the independent association of NLR with the severity of disease in schizophrenia. NLR was significantly decreased in schizophrenic patients after antipsychotic intervention. There is the discrepant correlation between NLR and psychiatric symptoms in schizophrenic patients with or without antipsychotic therapy. NLR is positively associated with the severity of disease in drug-free patients, whereas NLR is negatively associated with negative symptoms in drug-therapy patients. The findings confirm the hypothesis that NLR is independently associated with severe psychopathological symptoms in schizophrenia. This work supports the role of inflammation in psychotic illnesses and the effect of antipsychotic administration on NLR. These data suggest that NLR may be not only a useful inflammatory biomarker for assessment of disease severity in drug-free schizophrenic patients, but also a potential target to improve the course of the psychotic disorder. To investigate the role of specific antipsychotic medications in the relationship between NLR and the severity of disease, it is better to identify the patients who are receiving a single type of antipsychotic drug. We will investigate the unique mechanism of action of each specific antipsychotic medication in the future studies.

## Data Availability Statement

The raw data supporting the conclusions of this article will be made available by the authors, without undue reservation.

## Ethics Statement

The studies involving human participants were reviewed and approved by the ethics committee of Nanjing Medical University Affiliated Brain Hospital. Written informed consent for participation was not required for this study in accordance with the national legislation and the institutional requirements.

## Author's Note

This manuscript has been released as a pre-print at Research Square (43).

## Author Contributions

HS designed the study development program. XZ and XW collected the data from the Electronic Medical Record and Laboratory Information System for the analyses, wrote and revised the paper. RL and YX performed the statistical analyses. JY, HS, and WL critically reviewed and interpreted the data. The study was set up and conducted under the supervision of HS. All authors contributed to the article and approved the submitted version.

## Conflict of Interest

The authors declare that the research was conducted in the absence of any commercial or financial relationships that could be construed as a potential conflict of interest.
